# Evidence of Phytotoxicity and Genotoxicity *in Hordeum vulgare* L. Exposed to CeO_2_ and TiO_2_ Nanoparticles

**DOI:** 10.3389/fpls.2015.01043

**Published:** 2015-11-25

**Authors:** Alessandro Mattiello, Antonio Filippi, Filip Pošćić, Rita Musetti, Maria C. Salvatici, Cristiana Giordano, Massimo Vischi, Alberto Bertolini, Luca Marchiol

**Affiliations:** ^1^Department of Agriculture and Environmental Sciences, University of UdineUdine, Italy; ^2^Centro di Microscopie Elettroniche “Laura Bonzi”, Istituto di Chimica dei Composti OrganoMetallici, Consiglio Nazionale delle RicercheFirenze, Italy; ^3^Tree and Timber Institute, Istituto Per La Valorizzazione del Legno e delle Specie Arboree-CNRFirenze, Italy

**Keywords:** barley, cerium oxide nanoparticles, titanium oxide nanoparticles, genotoxicity, oxidative stress

## Abstract

Engineered nanoscale materials (ENMs) are considered emerging contaminants since they are perceived as a potential threat to the environment and the human health. The reactions of living organisms when exposed to metal nanoparticles (NPs) or NPs of different size are not well known. Very few studies on NPs–plant interactions have been published, so far. For this reason there is also great concern regarding the potential NPs impact to food safety. Early genotoxic and phytotoxic effects of cerium oxide NPs (*n*CeO_2_) and titanium dioxide NPs (*n*TiO_2_) were investigated in seedlings of *Hordeum vulgare* L. Caryopses were exposed to an aqueous dispersion of *n*CeO_2_ and *n*TiO_2_ at, respectively 0, 500, 1000, and 2000 mg l^-1^ for 7 days. Genotoxicity was studied by Randomly Amplified Polymorphism DNA (RAPDs) and mitotic index on root tip cells. Differences between treated and control plants were observed in RAPD banding patterns as well as at the chromosomal level with a reduction of cell divisions. At cellular level we monitored the oxidative stress of treated plants in terms of reactive oxygen species (ROS) generation and ATP content. Again *n*CeO_2_ influenced clearly these two physiological parameters, while *n*TiO_2_ were ineffective. In particular, the dose 500 mg l^-1^ showed the highest increase regarding both ROS generation and ATP content; the phenomenon were detectable, at different extent, both at root and shoot level. Total Ce and Ti concentration in seedlings was detected by ICP-OES. TEM EDSX microanalysis demonstrated the presence of aggregates of *n*CeO_2_ and *n*TiO_2_ within root cells of barley. *n*CeO_2_ induced modifications in the chromatin aggregation mode in the nuclei of both root and shoot cells.

## Introduction

It is estimated that by 2020 about six million people will be employed worldwide in industries that use nanotechnologies, which will have the potential to produce goods for a market of more than 3,000 billion dollars (Roco, 2011). There is therefore a tumultuous development of new materials justified by a rapid growth of technological and commercial applications. Model simulations demonstrated that flows of engineered nanomaterials (ENMs) are able to reach several natural ecosystems (Colman et al., 2013). Cerium oxide nanoparticles (NPs; *n*CeO_2_) and titanium oxide NPs (*n*TiO_2_) are among the top ten most produced ENMs by mass (Keller et al., 2013) and used in cosmetics industries, in solar cells, paints, cements, coatings, in agriculture and the food industry (Gogos et al., 2012; Piccinno et al., 2012; Parisi et al., 2015). *n*CeO_2_ and *n*TiO_2_ were included in the list of ENMs of priority for immediate testing by the Organization for Economic Cooperation and Development (OECD, 2010). From point sources (e.g., discharges of wastewaters from industries or landfills), such materials will tend to accumulate in sediments and soils, exposing the organisms inhabiting these environments to potential risks (Liu and Cohen, 2014).

Plants are able to assimilate metal nanoparticles (MeNPs) largely depending on the type of plant and the size of MeNPs (Rico et al., 2011). In addition, the primary particle size of MeNPs is relevant for their bioavailability and therefore their toxicity (Van Hoecke et al., 2009), also raising questions on the potential for MeNPs exposure of crops and food safety (Hong et al., 2013). Experimental evidences were reported by Zhang et al. (2011) which studied the *n*CeO_2_ uptake and translocation in cucumber, reporting an higher Ce assimilation in plants treated with 7 nm Ce than 25 nm ones. Clément et al. (2013) reported similar results for *n*TiO_2_ on rapeseed plantlets treated with 14–25 nm particles. Another functional property that influences the MeNPs plant assimilation is the agglomeration/aggregation status that in turn is influenced directly by the zeta-potential (Navarro et al., 2008). Song et al. (2013a) demonstrated a negative correlation between *n*TiO_2_ agglomeration/aggregation and assimilation in tomato. A similar behavior could be hypothesized also for *n*CeO_2_.

Although there are potential positive applications of ENMs in agriculture (Parisi et al., 2015), studies on the toxicity of MeNPs have shown early negative consequences on crops due to genotoxic and phytotoxic effects (Miralles et al., 2012; Gardea-Torresdey et al., 2014). From an ecological point of view, this raises questions about potential risks due to the input of MeNPs in the food chain. Early plant MeNPs toxicity can be measured observing seed germination, root elongation, DNA mutations (López-Moreno et al., 2010a; Atha et al., 2012) or changes in biochemical parameters (Rico et al., 2013; Schwabe et al., 2013).

The aims of this study were to determine the early phytotoxic and genotoxic effects of *n*CeO_2_ and *nTi*O_2_ on barley (*Hordeum vulgare* L.) plants. The FAO ranks barley fourth in the top five cereals in the world ordered based on production tonnage (FAOSTAT, 2014) and the cereal is one of the major crops grown worldwide for human and animal consumption. Suspensions of *n*CeO_2_ and *n*TiO_2_ were prepared at 0, 500, 1000, and 2000 mg l^-1^. Phytotoxicity of NPs was determined through percentage of germination and root elongation, ATP and ROS generation in root and leaf cells. Genotoxicity was investigated by the mitotic index and RAPDs. Ce and Ti uptake and translocation within seedling tissues were determined by inductively coupled plasma-optical emission spectroscopy (ICP-OES), while *n*CeO_2_ and *n*TiO_2_ within plant cells were detected by transmission electronic microscope and energy dispersive x-ray spectrometer (TEM–EDX).

## Materials and Methods

### Nanoparticles Characterization

The cerium^(IV )^ oxide (*n*CeO_2_) and titanium^(IV )^ oxide anatase (*n*TiO_2_) powders with a nominal average particle size <25 nm were purchased from Sigma–Aldrich (Milwaukee, WI, USA). The specific surface area of the *n*CeO_2_ and *n*TiO_2_ was measured by the Brunauer–Emmett–Teller (BET) method by using the Surface Area and Pore Size Analyzer SA 3100 plus (Beckman Coulter, USA).

The *n*CeO_2_ and *n*TiO_2_ powders were suspended in deionized water at a concentration of 1000 mg l^-1^ and sonicated at 60°C for 30 min. The suspensions were characterized for Z-average size, measured as hydrodynamic diameter, zeta potential, via electrophoretic mobility, and polydispersity index (PDI), calculated from the signal intensity, by the dynamic light scattering (DLS) method using the Nano ZS90 (Malvern Instruments, UK). The *n*CeO_2_ and *n*TiO_2_ powder suspensions at three different concentrations (500, 1000, and 2000 mg l^-1^) were prepared in MilliQ^®^ water by sonication for 30 min at room temperature and then stirred for 15 min. The range of concentrations (0, 500, 1000, and 2000 mg L^-1^) was chosen according to Yang and Watts (2005), Lin and Xing (2007), and López-Moreno et al. (2010a).

### Seed Germination and Root Elongation

Caryopses of *H. vulgare* L. var. Tunika were provided by S.I.S. Società Italiana Sementi (San Lazzaro di Savena, Bologna, Italy). The caryopses were sterilized by orbital agitation with 70% ethanol for 2 min and then with 5% sodium hypochlorite plus some drops of Tween 80 for 30 min. They were rinsed six times with sterilized MilliQ^®^ water. All caryopses were transferred in sterile conditions into 15 mm Petri dishes containing filter paper (Ø 90 mm Whatman No. 1) soaked with 8 ml of MilliQ^®^ water (control treatment) or 8 ml of *n*CeO_2_ or *n*TiO_2_ suspensions at different concentrations. The Petri dishes were taped and placed in the dark at 21°C for 3 days. The germination percentage was calculated as the ratio of germinated seeds out the total seeds of each Petri dish. A second set of caryopses were treated for 7 days in the same conditions to evaluate root elongation and Ce and Ti uptake. The seedlings were photographed and Image J software (Schneider et al., 2012) was used to measure roots length. Root elongation was calculated as the average or the sum of all roots emerged from each seed. The experiments were performed in triplicate.

### Mitotic Index

The germinated seedlings with actively growing roots (2.5 cm in length) were placed in the *n*CeO_2_ and *n*TiO_2_ suspensions (0, 500, 1000, 2000 mg l^-1^) for 24 h. After treatment the root tips were fixed in 3:1 alcohol : acetic acid and then, kept in 70% ethanol at 4°C. The root tips were rinsed in deionized water for 5 min, hydrolyzed in 1N HCl for 8 min at 60°C, rinsed in deionized water for 5 min, stained in leuco-basic-fuchsine for 45 min and washed in tap water for 5 min. The root tips were then transferred to 45% acetic acid for 1 to 5 min, root caps were removed, and the roots were dissected to release the meristematic cells. Ten tips per treatment were evaluated and each treatment was replicated three times, for a total of about 10,000 cell observations. The mitotic index was evaluated in Feulgen stained preparations as the percentage of dividing cells out of the total number of cells scored.

### Random Amplified Polymorphic DNA (RAPD) Analysis

The genotoxicity of *n*CeO_2_ and *n*TiO_2_ was investigated by observing the band profile after a random amplified polymorphic DNA (RAPD) assay on six replicates per treatment obtained from seedlings exposed as for mitotic index experiment. Plant genomic DNA was extracted from root tips using the DNeasy Plant Mini Kit (QIAGEN^®^) according to manufacturer’s protocol. PCR reactions were performed with 30 ng of genomic DNA as a template using six primer pairs: OPA04 (AATCGGGCTG), OPA10 (GTGATCGCAG), OPB01 (GTTTCGCTCC), OPB03 (CATCCCCCTG), OPB12 (CCTTGACGCA), and OPB20 (GGACCCTTAC). The PCR conditions consisted of an initial Taq polymerase activation at 95°C for 5 min, followed by 45 cycles of denaturation (95°C, 1 min), annealing (35°C, 1 min), and extension (72°C, 1 min) with a final extension for 10 min at 72°C. The PCR products were subjected to electrophoresis on 1.6% agarose in TBE 0.5%, for 2 h at 60 V/cm stained with GelRed^®^ and photographed for band scoring.

### Evaluation of ATP Content

ATP content was determined by means of the luciferin–luciferase luminometric assay (Lundin, 1984). Root and shoot of each seedling were separated, frozen with liquid nitrogen and ground to a fine powder. Tissue powder (100 ± 20 mg FW) was suspended in 1 ml of 50 mM Tris-HCl (pH 7.5), 0.05% (w/v) Triton X-100 and immediately kept at 95°C for 3 min to inactivate any possible hydrolytic activity. After cooling, samples were centrifuged to obtain the cellular soluble fraction in the supernatant. The sample assays were performed in a 96-well plate with ATPlite Luminescence ATP Detection Assay System, (PerkinElmer) according to manufacturer’s protocol. Aliquots (20 μl) of soluble fraction were mixed with 20 μl of ATPlite buffer in 130 μl of 50 mM Tris-HCl (pH 7.5) and 5 mM MgCl_2_. Signals were detected by a Multilabel Counter (WALLAC, model 1420, PerkinElmer, Waltham, MA, USA). Actual ATP concentration of each experiment (expressed as nmol ATP g^-1^ f. w.) was calculated by a calibration curve obtained with commercially purchased ATP (Sigma, USA) in a 8–100 nM range.

### Reactive Oxygen Species (ROS) Determination

The generation of ROS was monitored by the method of Wang and Joseph (1999), using 2′,7′-dichlorodihydrofluorescein diacetate (H_2_DCFDA) as a probe. Tissue powder (0.5 g f. w.) obtained from both roots and shoots was extracted in 2.5 ml cold acetone and incubated for 4 h at 4°C. After centrifugation at 1000 *g* for 10 min, the pellet was homogenized in 1 ml of 50 mM Tris-HCl (pH 7.5), 0.4 M sucrose and 1 mM EDTA by Turrax device. The sample was again centrifuged for 15 min and the supernatant stored at –80°C until analysis. Aliquots of sample (20 μl) were incubated in 96-well microplate with 5 μM H_2_DCFDA and 180 μl of 50 mM Tris-HCl (pH 7.5). Detection was performed by fluorimetric assay using Multilabel Counter (WALLAC, model 1420, PerkinElmer) with orbital shaking and reading for 1.75 h at 5 min intervals with excitation filter set at 485 ± 10 nm and the emission filter set at 530 ± 10 nm. Values of relative fluorescence (RFU) were expressed as RFU mg^-1^ protein. Protein concentration was estimated by the Bradford (1976) method.

### Cerium and Titanium in Seedling Tissues

The seedlings were washed by agitation with 0.01 M HNO_3_ for 30 min and rinsed three times by agitation with MilliQ^®^ water for 15 min. The seedling roots and shoots were then oven-dried at 105°C for 24 h and 0.5 g material was digested using 10 ml of HNO_3_ in a microwave oven (CEM, MARS Xpress) according to the USEPA 3052 method (USEPA, 1995). After mineralization, the plant extracts were filtered (0.2 μm PTFE), diluted and analyzed. Total content of Ce and Ti was determined by an ICP-OES (Varian Inc., Vista MPX). The accuracy of the analytical procedure adopted for ICP-OES analysis was checked by running standard solutions every 20 samples. Yttrium was used as the internal standard.

### TEM Observations and X-ray Microanalysis

The morphology of NPs was assessed by direct observation of suspension of *n*CeO_2_ or *n*TiO_2_ NPs under the TEM. Drops of suspensions (prepared as described above) were placed on carbon–formvar coated nickel grids, dried at room temperature and observed under a Philips CM 10 (FEI, Eindhoven, The Netherlands) TEM, operating at 80 kV.

For microscopic analyses *in planta*, tissues from seedlings treated with *n*CO_2_ or *n*TiO_2_ at 1000 and 2000 mg l^-1^ were sampled as in the root elongation experiment were sampled. Roots and shoots were excised, cut into small portions (2 mm × 3 mm) and fixed for 2 h at 4°C in 0.1% (w/vol) buffered sodium phosphate and 3% (w/v) glutaraldehyde at pH 7.2. They were then post-fixed with 1% osmium tetroxide (w/v) in the same buffer for 2 h, dehydrated in an ethanol series, and embedded in Epon/Araldite epoxy resin (Electron Microscopy Sciences, Fort Washington, PA, USA). For conventional TEM observations, serial ultrathin sections from embedded leaf tissues were cut with a diamond knife, mounted on uncoated 200 mesh copper grids (Electron Microscopy Sciences, Fort Washington, PA, USA), stained in uranyl acetate and lead citrate, and then observed under TEM as reported above.

For X-ray microanalysis, unstained ultrathin sections were placed on formvar/carbon-coated 200 mesh nickel grids and dried at room temperature. The nature of NPs observed in plant tissues was determined by a TEM (PHILIPS CM 12, FEI, Eindhoven, The Netherlands) equipped with an EDS-X-ray microanalysis system (EDAX, AMETEK, Mahwah NJ, USA, software EDAX Genesis). The images were recorded by a Megaview G2 CCD Camera (Olympus; software iTEM FEI, Analysis Image Processing).

### Data Analysis

One-way analysis of variance (ANOVA) was conducted to test differences in the plants’ behavior. Tukey’s Multiple Comparison test at 0.05 *p* level were used to compare means. Statistical analyses were performed using the SPSS program (SPSS Inc., Chicago, IL, USA, ver. 17). Principal Coordinate Analysis (PCoA) was computed based on the binary genetic distance option in GenAlEx v. 6.501 software (Peakall and Smouse, 2012). Graphics were produced using CoPlot (CoHort ver. 6.204, Monterey, CA, USA).

## Results

### Nanoparticles Characterization

The specific surface values obtained by BET measurements were 46.1 m^2^ g^-1^ for *n*CeO_2_ and 61.6 m^2^ g^-1^ for *n*TiO_2_. The Z-average sizes of the *n*CeO_2_ and *n*TiO_2_ suspended in deionized water were 174 ± 1.2 nm and 925 ± 105 nm, respectively, these values result remarkable higher respect the declared producer dimensions. The zeta potentials were 0.027 ± 0.064 mV for *n*CeO_2_ and 19.9 ± 0.55 mV *n*TiO_2_. These parameter values put in evidence their instability, in fact for both NP types are included in the range of the NP instability (–30 mV ÷ +30 mV) and justify the differences between the declared dimension and the measured ones. The PDI of *n*CeO_2_ and *n*TiO_2_ were 0.339 ± 0.011 and 0.841 ± 0.173, respectively. These values indicate a narrow dimensional distribution of *n*CeO_2_ respect to *n*TiO_2_.

### Caryopses Germination and Root Elongation

Effects of *n*CeO_2_ and *n*TiO_2_ on caryopses germination and root growth are shown in **Table 1**. Since there was not a statistically significant effect of concentrations for *n*CeO_2_ and *n*TiO_2_, our results demonstrate that, even at the highest level of concentration, caryopses germination is not affected by *n*CeO_2_ or *n*TiO_2_ (**Table 1**). At the end of our experiment the barley seedlings had reached coleoptile emergence. At this stage typically has between six and seven seminal roots (Knipfer and Fricke, 2011). In our experiment the number of seminal roots was not affected by *n*CeO_2_ and *n*TiO_2_ (**Table 1**). On the contrary, in both cases the development of root tissues was influenced in a similar manner by the treatments. In fact, there was a significant effect of both *n*CeO_2_ (*p* < 0.05) and *n*TiO_2_ (*p* < 0.05) on the average length of the seminal roots. *Post hoc* comparison tests indicated that root elongation in seedlings treated with 500 mg l^-1^ *n*CeO_2_ and *n*TiO_2_ was significantly lower than controls (–24.5 and –14.8%, respectively). At higher *n*CeO_2_ and *n*TiO_2_ concentrations we would have expected to see a further reduction in the development of seminal roots. However, this did not occur since the average length of seminal roots was similar to controls (**Table 1**).

**Table 1:** Germination percentage of seeds, number of seminal roots and root length in barley seedlings treated with 0, 500, 100, and 2000 mg l^-1^ of *n*CeO_2_ and *n*TiO_2_.

Treatment	*n*CeO_2_			*n*TiO_2_		
		
	Germination (%)	Seminal roots (*n*)	Root length (mm)	Germination (%)	Seminal roots (*n*)	Root length (mm)
Ctrl	87 ± 1.76 a	5.2 ± 0.18 a	52.7 ± 4.13 a	88 ± 1.20 a	6.6 ± 0.34 a	53.3 ± 3.03 a
500 mg l^-1^	83 ± 2.03 a	5.5 ± 0.22 a	39.8 ± 2.24 b	87 ± 1.76 a	6.1 ± 0.27 a	45.4 ± 2.85 b
1000 mg l^-1^	80 ± 2.08 a	5.2 ± 0.26 a	45.8 ± 17.8 ab	85 ± 1.45 a	6.5 ± 0.22 a	53.9 ± 3.13 a
2000 mg l^-1^	79 ± 1.86 a	4.9 ± 0.25 a	43.8 ± 1.72 ab	87 ± 1.76 a	6.4 ± 0.13 a	58.5 ± 2.97 a


*Values are mean ± SE (*n* = 3). Different letters indicate statistical difference between treatments at Tuckey’s test (*p* < 0.05).*

### Cerium and Titanium in Plant Tissues

Although without visible symptoms of phytotoxicity, the concentration of total Ce and Ti in the tissues of barley seedlings showed (i) a dose-response and (ii) a different magnitude of accumulation between Ce and Ti. **Table 2** shows the concentration of Ce and Ti in the fractions of barley seedlings. As expected Ce and Ti accumulated much more within root tissues than in the shoot (*p* < 0.05). Ce concentration in the roots increased significantly (*p* < 0.05) as the concentration of *n*CeO_2_ in the growth medium increased (**Table 2**). A statistically significant effect of treatments in Ce accumulation in the shoots (*p* < 0.001) was verified. Mean comparisons showed differences among the treatments. Ce concentration in shoots did not significantly differ between the 500 and 1000 mg l^-1^ Ce treatment (38.3 and 98.1 mg Ce kg^-1^ DW, respectively), whereas at 2000 mg *n*CeO_2_ L^-1^ a Ce concentration of 622 mg Ce kg^-1^ DW was observed in the shoots, which is significantly different from other values (**Table 2**).

**Table 2:** Concentration of total Ce and Ti in roots and shoots of barley seedlings treated with 0, 500, 100, and 2000 mg l^-1^ of *n*CeO_2_ and *n*TiO_2_.

Treatment	Ce roots (mg kg^-1^ DW)	Ce coleoptile (mg kg^-1^ DW)	Ti roots (mg kg^-1^ DW)	Ti coleoptile (mg kg^-1^ DW)
Ctrl	<d.l.	<d.l.	<d.l.	<d.l.
500 mg l^-1^	579 ± 168 b	38.3 ± 5.77 b	<d.l.	<d.l.
1000 mg l^-1^	5262 ± 1751 b	98.1 ± 40.2 b	35.2 ± 17.3 b	7.83 ± 3.3 b
2000 mg l^-1^	20,714 ± 5722 a	622 ± 95.1 a	412 ± 127 a	26.2 ± 8.71 a


*Values are mean ± SE (*n* = 3). Different letters indicate statistical difference between treatments at Tuckey’s test (*p* < 0.05).*

Titanium concentrations in barley roots and shoots were one–two orders of magnitude lower compared to Ce. However, also in this case a statistically significant dose dependent increase was also observed. With the lowest *n*TiO_2_ treatment (500 mg l^-1^) Ti concentration in roots was negligible and no Ti was detected in shoots (**Table 2**). At the intermediate *n*TiO_2_ treatment (1000 mg l^-1^) the root tissues had 37.2 mg Ti kg^-1^ DW which is significantly lower (*p* = 0.0001) than 413 mg Ti kg^-1^ DW found at highest *n*TiO_2_ treatment (**Table 2**). Finally, we verified that also Ti concentration in the shoots also responded positively to the treatments (*p* < 0.001). The mean Ti concentration detected in barley shoots were 7.83 mg kg^-1^ DW and 26.2 mg kg^-1^ DW for 1000 and 2000 mg nTiO_2_ l^-1^, respectively (**Table 2**).

### Ce and Ti Nano-aggregates in Plant Tissues

**FIGURE 1:**
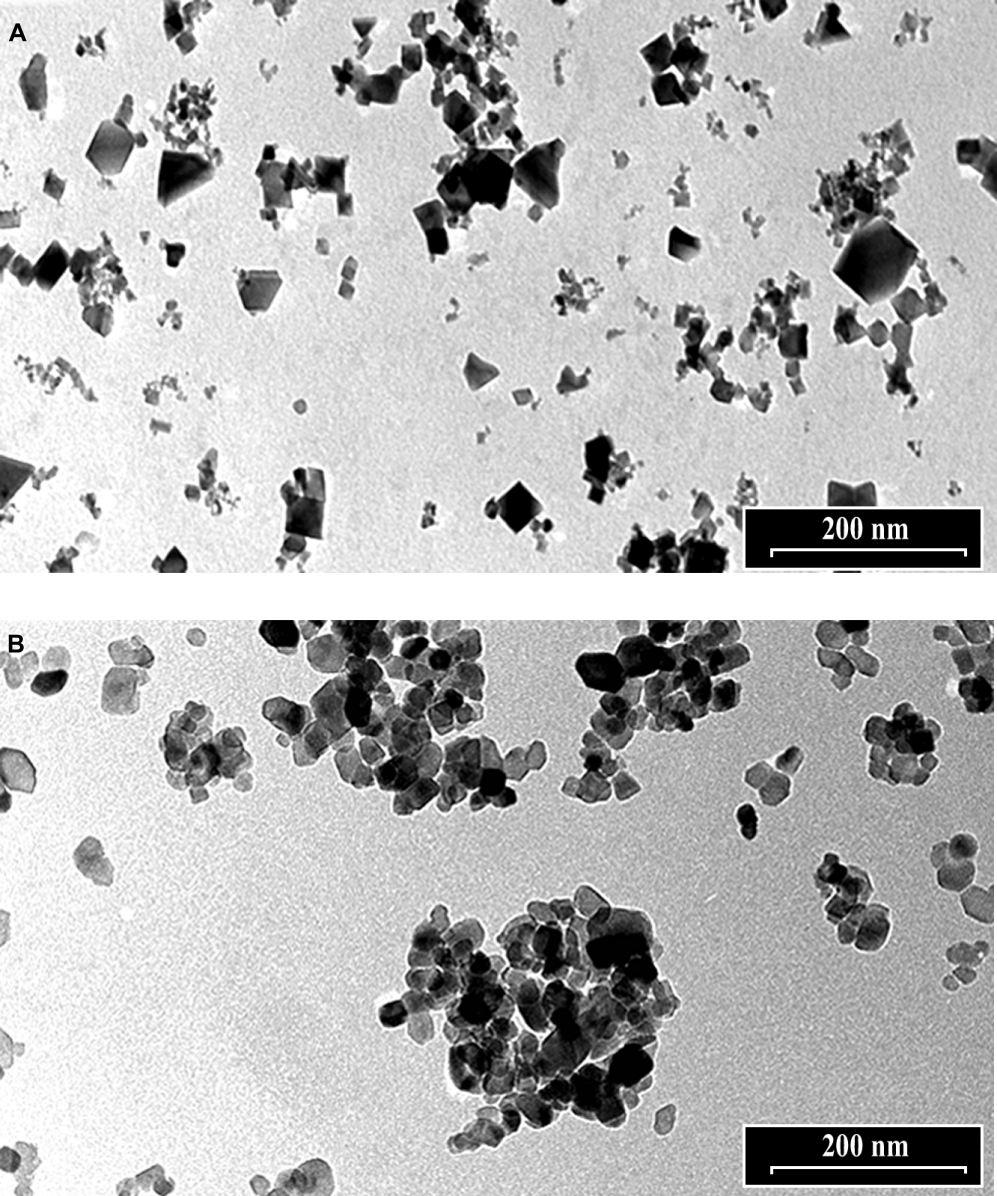
**Transmission electron microscope images of 1000 mg l^-1^ suspensions of **(A)***n*CeO_2_ and **(B)***n*TiO_2_**.

**FIGURE 2:**
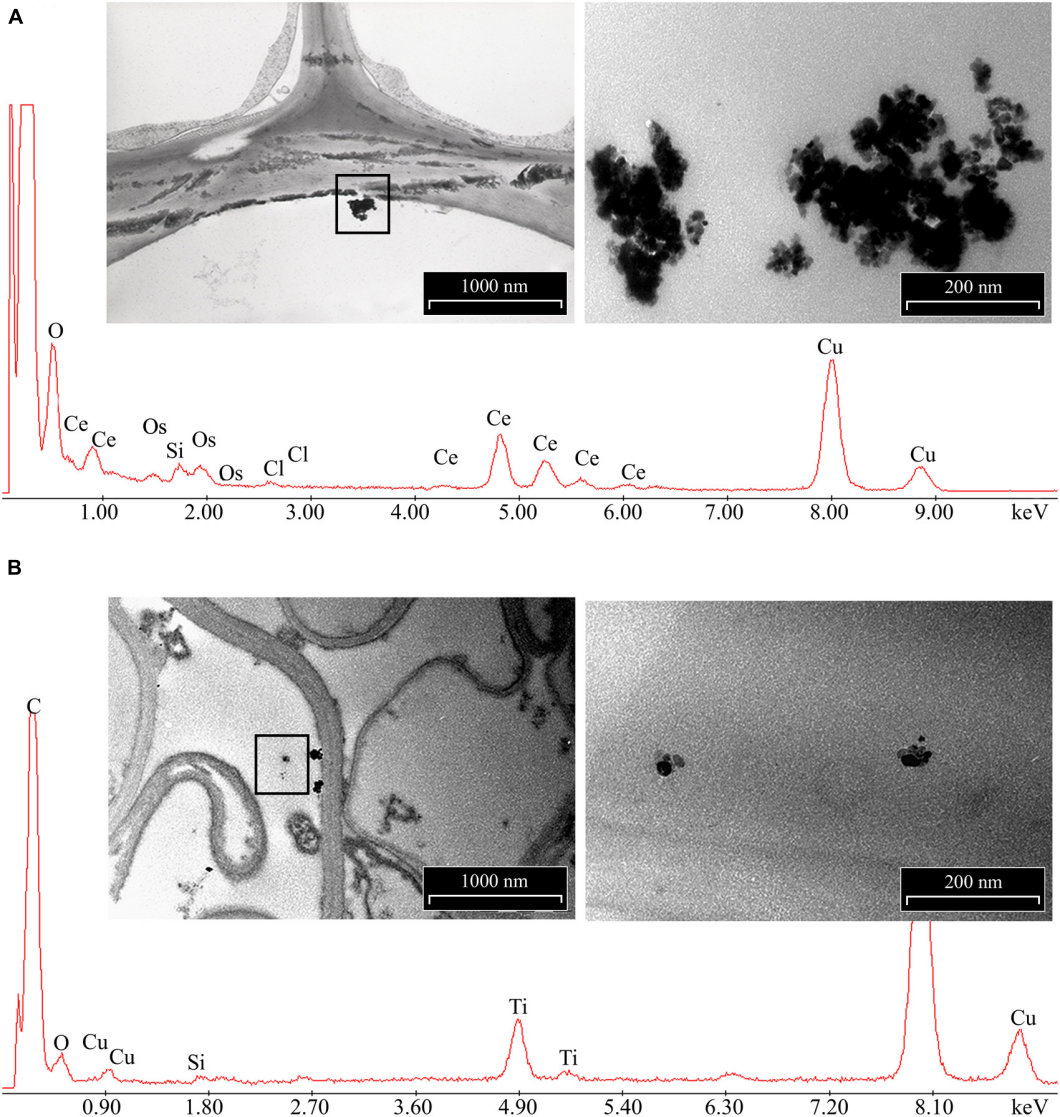
**Representative images of electron dense precipitates recovered in root tissues of *Hordeum vulgare* exposed to 1000 mg l^-1^ of **(A)***n*CeO_2_ and **(B)***n*TiO_2_ and X-ray spectra of elements recovered in.** Insets represent enlarged regions where X-ray microanalyses have been performed. Presence of C, Os were due to sample preparation, Cu to the grids used as section support.

The morphology of *n*CeO_2_ and *n*TiO_2_ NPs is visible in **Figures 1A,B**, respectively. Transmission electron microscopy analysis demonstrated that CeO_2_ particles exhibited an approximate equi-axes shape with sharp edges (**Figure 1A**), while particle sharp edges are less evident in TiO_2_. To assess the possible uptake of *n*CeO_2_ or *n*TiO_2_ from the culture medium to the root tissues and the translocation to the different parts of the plantlets, we performed ultrastructural analyses on roots and shoot tissues. Several clusters of NPs were found in cortical parenchymal tissues of roots, both in the case of *n*CeO_2_ (**Figure 2A**) and *n*TiO_2_ treatment, at all concentrations. Clusters were also observed in the xylem, even if in to lesser extent (**Figure 2B**). EDS-X ray microanalysis allowed the identification of the clusters as aggregates of Ce and Ti nanoparticles.

**FIGURE 3:**
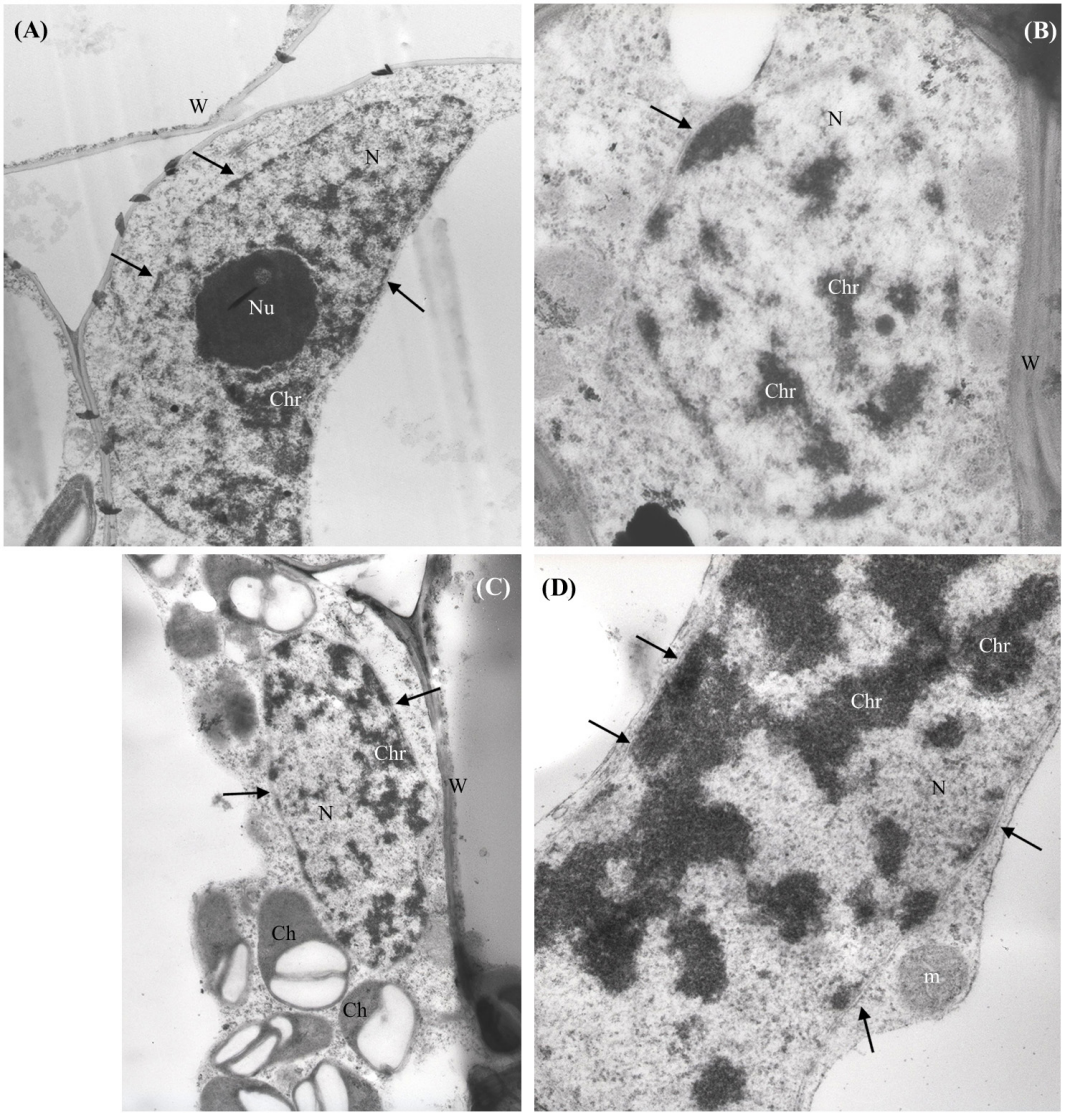
**Representative micrographs of nuclei (N) from shoot **(A–C)** and root **(D)** parenchymal cells of *Hordeum vulgare*.** **(A)** Control untreated shoot: nucleus presents regular shape, nuclear membranes are intact (arrows), nucleolus (Nu) and chromatin (Chr) appear normally dispersed. **(B,C)** 1000 mg *n*CeO_2_ l^-1^- treated shoot and **(D)**, 1000 mg *n*CeO_2_ l^-1^- treated root: nuclei still present normal shape and apparently undamaged membranes, while chromatin shows condensation. Ch, chloroplasts; m, mitochondrion.

No NPs were detected in the shoots of *n*CeO_2_ or *n*TiO_2_ treated plantlets. The ultrastructure of all observed tissues appeared preserved. No necrosis or damage to membranes, nor cell modifications were detected. In general, the cell compartments were not significantly affected by treatments, except for the nuclei of parenchymal cells of root and shoot of seedlings treated with *n*CeO_2_ (1000 and 2000 mg l^-1^), which showed compact chromatin (**Figures 3A–D**).

### ATP and ROS

**FIGURE 4:**
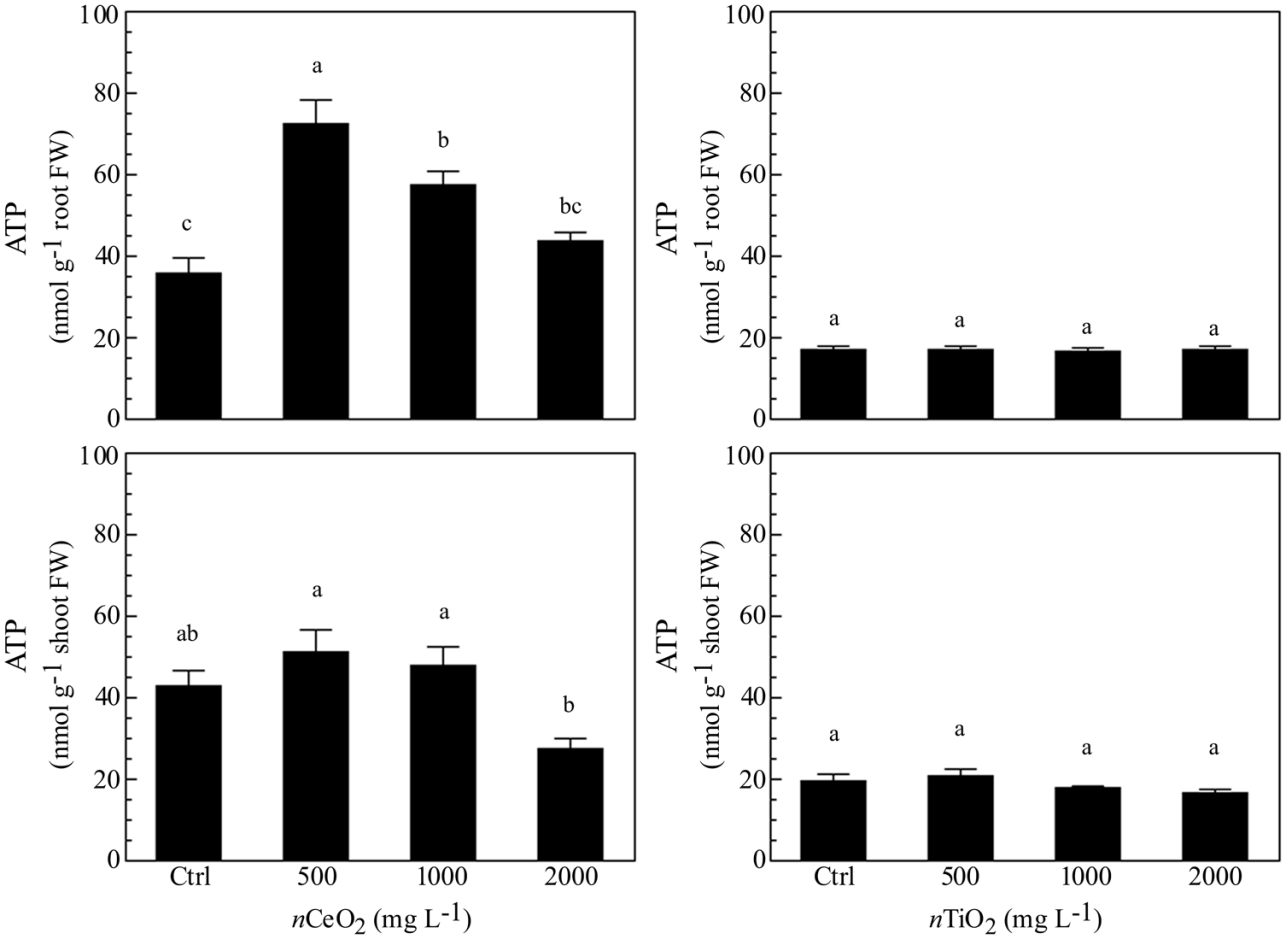
**Determination of ATP concentration in extracts obtained from plantlets of barley roots and shoots, grown on wet paper filters, in the presence of different concentrations of *n*CeO_2_ and *n*TiO_2_**.

The evaluation of ATP concentration aimed to evidence the energetic status in different fractions of barley seedlings exposed to *n*CeO_2_ and *n*TiO_2_. The different concentrations of *n*CeO_2_ induced a statistically significant effect (**Figure 4**), with a trend of values peaking at 500 and 1000 mg l^-1^, in root and lowering at 2000 mg l^-1^ in shoot samples. The highest *n*CeO_2_ (2000 mg l^-1^) reached a low concentration of ATP in roots, statistically comparable to control samples. On the contrary, *n*TiO_2_ induce no significant changes of ATP concentration, since different *n*TiO_2_ doses were similar to the controls in both roots and shoots (**Figure 4**).

**FIGURE 5:**
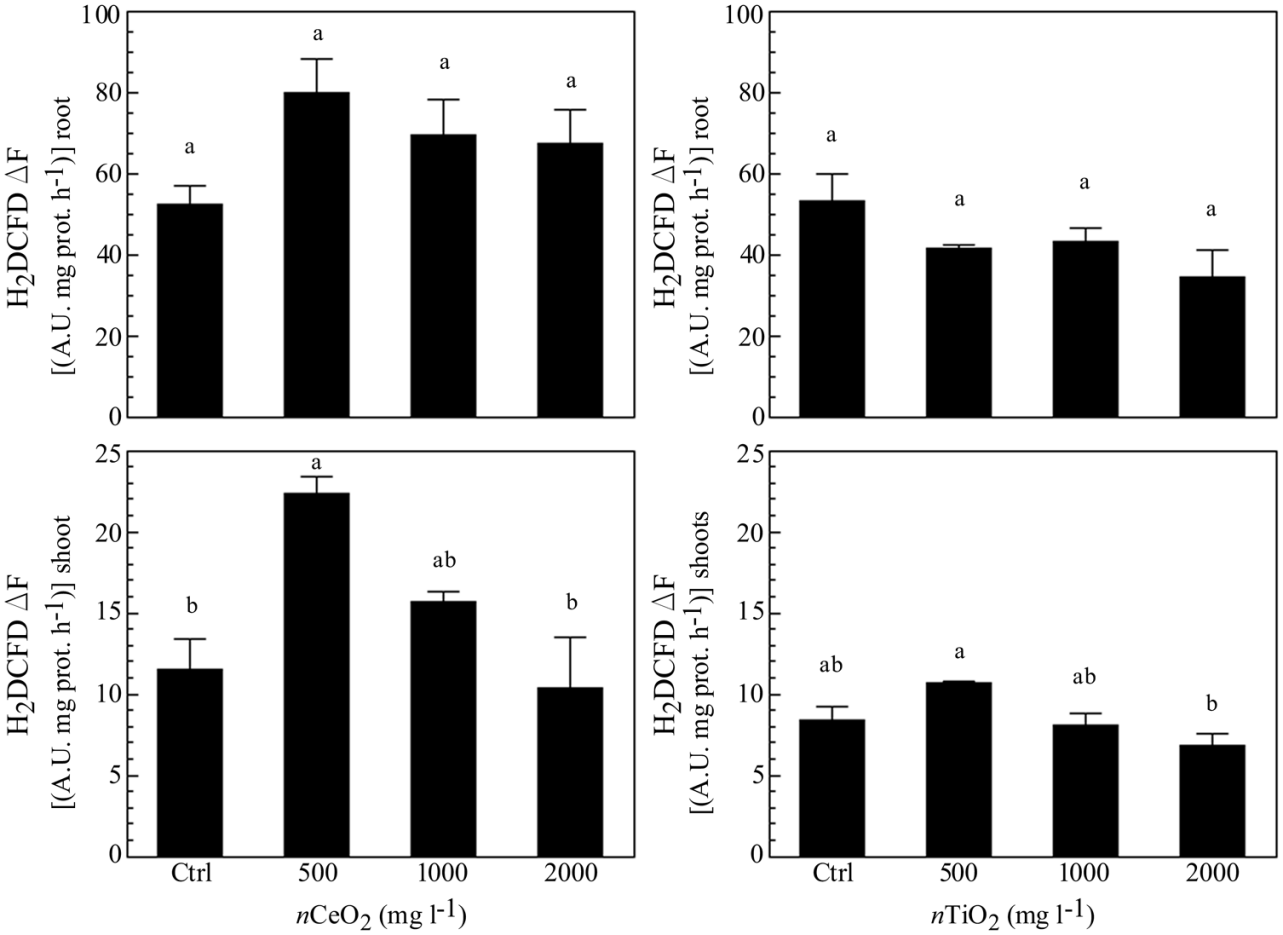
**Evaluation of reactive oxygen species (ROS) evolution in extracts obtained from plantlets of barley roots and shoots, grown on wet paper filters, in the presence of different concentrations of *n*CeO_2_ and *n*TiO_2_.** The analysis was performed by means of a fluorimetric probe.

The measurement of ROS was performed as marker for oxidative stress. Similarly to ATP content, *n*CeO_2_ were able to induce an increase of a ROS formation at all the concentrations assayed (**Figure 5**), in comparison with the control, although no statistically significant differences were observed. Also for this parameter, a trend with a peak at 500 mg l^-1^ was present in both roots and shoots. In the case of *n*TiO_2_ (**Figure 5**), the treatments did not show any difference, if compared with the control in roots, whereas a decrease of ROS level was observed at the higher dose (2000 mg l^-1^) in shoots.

### Mitotic Index and RAPDs

**FIGURE 6:**
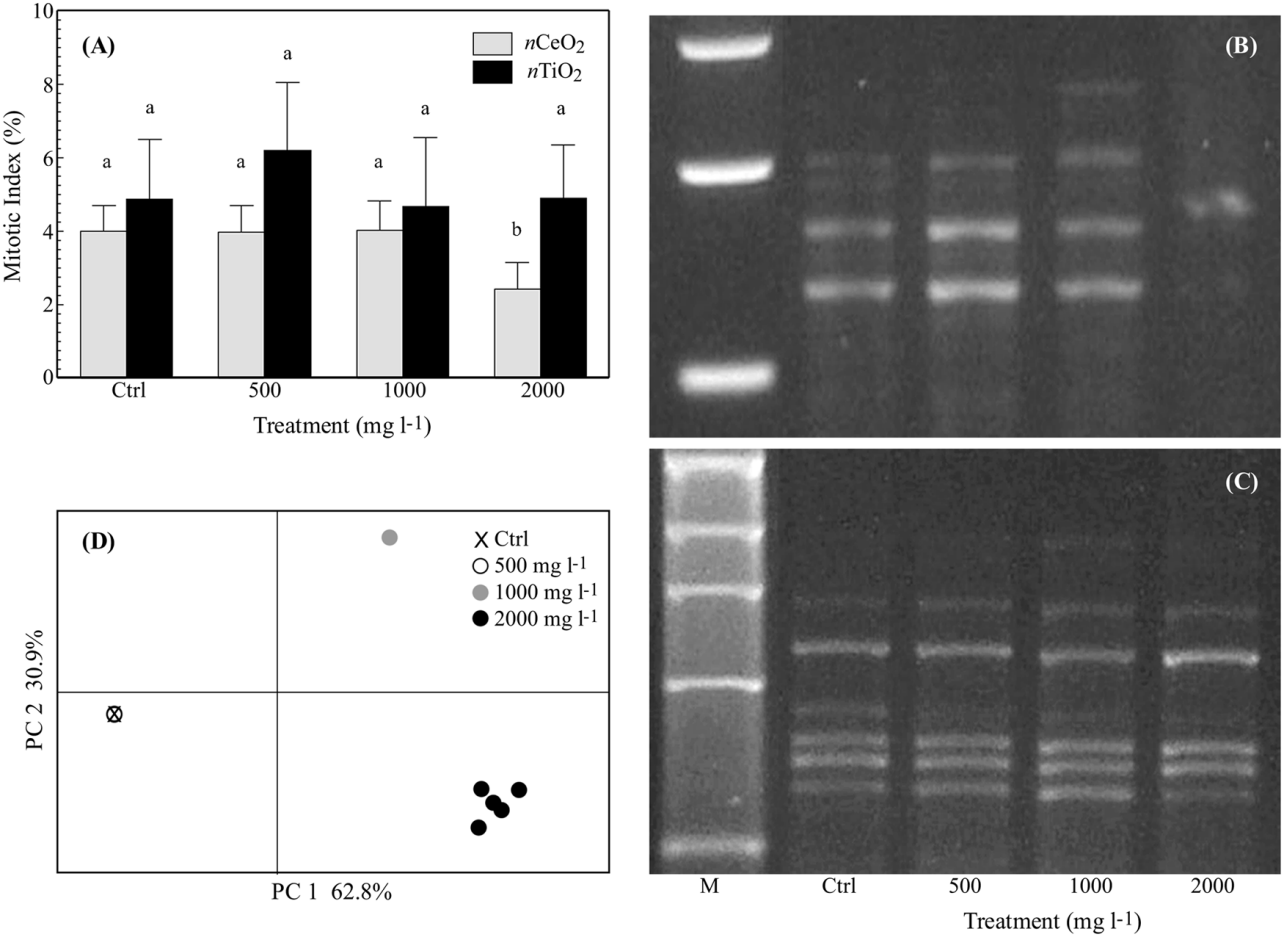
**(A)** Mitotic index (%; mean ± SE) observed in root tips of seedlings of barley treated with 0–2000 mg l^-1^ of *n*CeO_2_ and *n*TiO_2_. Different letters indicate statistical difference between treatments at Tuckey’s test (*p* < 0.05). **(B,C)** Representative RAPD profiles from the roots of barley seedlings treated with *n*CeO_2_ **(B)** or *n*TiO_2_ **(C)** at control, 500-2000 mg l^-1^. The shown RAPD profiles were generated using primer OPA04 for *n*CeO_2_ (it is shown an enlargement around polymorphic zone) and OPB12 for *n*TiO_2_. The first line is a 1 kb DNA marker (M). **(D)** Principal coordinate analysis (PCoA) based on RAPD profiles from the barley roots with *n*CeO_2_. Values on axes indicate the variance explained.

The mitotic index was significantly reduced by *n*CeO_2_ 2000 mg l^-1^ (from 4 ± 1.2% in the control to 2.4 ± 1.2%). Instead, the *n*CeO_2_ 500 and 1000 mg l^-1^ treatments with mean values of 4 ± 1.3% were very similar to the control (**Figure 6A**). The treatments with *n*TiO_2_ with values of 6.2 ± 3.2%, 4.6 ± 3.2%, 4.9 ± 2.5% for the concentration at 500, 1000, and 2000 mg l^-1^, respectively, were not significantly different from the control (4.9 ± 2.8%; **Figure 6A**).

The six primers used for the RAPD analysis amplified for a total of 40 representative bands in controls with a variable number of 3 to 9 (9, 5, 6, 3, 9, 8, bands, respectively, for OPA04, OPB01, OPA10, OPB20, OPB12, and OPB03). Amplification was highly reproducible since the same RAPD profile was observed within control replicates. A concentration effect was observed for the *n*CeO_2_ treatments on the RAPD profiles. The same banding pattern as controls was obtained for the *n*CeO_2_ 500 mg l^-1^ treatment, whereas new profiles at 1000 mg l^-1^ were observed and three additional bands appeared and eight disappeared. Even greater variability was observed at *n*CeO_2_ 2000 mg l^-1^ with a total of 20 differences (appearing and disappearing bands) in treated plants (**Figures 6B,C**). The results were summarized by Principal Coordinates Analysis (PCoA), with almost 94% of the total variability explained by the two axes (**Figure 6D**). The overlap of the control and 500 mg l^-1^ treatments is notable, while the treatments at 1000 and 2000 mg l^-1^ are well separated in different quadrants. The band polymorphism in the different replicates at the higher concentration (2000 mg l^-1^) can be noticed by the point cloud (**Figure 6D**). In a similar way to what was observed fro the mitotic index, the *n*TiO_2_ treatments at each concentration have no effect on the RAPD profiles (**Figure 6D**).

## Discussion

Since plant nanotoxicology is a new field of investigation, specific ecotoxicological methods for the estimation of toxicity of ENPs have not yet been developed (Jośko and Oleszczuk, 2014). According to OECD guidelines, the acute effects of MeNPs on plant physiology are currently investigated by adapting the methods already used for traditional contaminants (Kühnel and Nickel, 2014). Evidence of MeNPs plant uptake and toxicity are still scarce and contradictory (Etheridge et al., 2013). This is likely because, compared to their bulk counterparts, MeNPs show particular properties, which are subjected to transformations (e.g., redox reactions, aggregation or agglomeration, and dissolution) according to different environmental factors. These changes might modify the ecotoxicological properties of MeNPs and thus, their interactions with the biota (Nowack et al., 2012; Maurer-Jones et al., 2013). However, despite these limitations, the experimental results obtained so far offer early indications on MeNPs phytotoxicity (Li et al., 2015; Rico et al., 2015a). Our data suggests that also in very simple experimental conditions, *n*TiO_2_, as expected taking into account their intrinsic properties, forms bigger agglomerates with a wider dimensional distribution than *n*CeO_2_.

### *n*CeO_2_ and *n*TiO_2_ Affects Seed Germination and Seedling Development

Previous studies carried out in controlled conditions reported that the toxicity of MeNPs in the early stages of plant growth is likely due to the following factors: (i) chemical and physical properties which influence the release of ions or the aggregation of particles in more stable forms and (ii) the size and shape of the particles, which determine the specific surface area of MeNPs (Yang and Watts, 2005; Lin and Xing, 2007).

In agreement with Rico et al. (2015b), we found that germination of barley was unaffected by 500–2000 mg l^-1^ *n*CeO_2_. This is in contrast with the results provided by López-Moreno et al. (2010a) who reported that suspensions of 2000 mg l^-1^ *n*CeO_2_ significantly reduced seed germination in maize, cucumber, tomato, and soybean. Possible explanations could be the greater Ce tolerance of barley to the treatment if compared to other species and/or to the very small size of Ce NPs they used (7 nm). Another explanation could be related to the chemical and physical properties of *n*CeO_2_, in particular his zeta potential value. This parameter is the cause of the agglomeration behavior of the *n*CeO_2_ that brings to a low bioavailability and the absence of phytotoxic effects on the treated seeds regards the germination percentage.

Another important issue that plays a role on seed/NP interaction, is the methodology adopted for seed treatment. In fact, following Lin and Xing (2007), we prepared the barley seeds for germination trials by soaking them in distilled water before starting treatments, whereas López-Moreno et al. (2010a) soaked the seeds directly in the *n*CeO_2_ suspensions. This different experimental approach could result in a different exposure of germinating seeds to *n*CeO_2_.

As regards Ti, there is a substantial agreement in literature on the fact that suspensions of *n*TiO_2_ do not affect seed germination, with few exceptions, as reported by Zheng et al. (2005) and Feizi et al. (2012). Our results are in accordance with those reported by other authors on rice, lettuce, radish, cucumber, tomato, and pea (Boonyanitipong et al., 2011; Wu et al., 2012; Song et al., 2013a; Fan et al., 2014).

Besides the germination percentage, we observed a negative influence of the treatments with *n*CeO_2_ and *n*TiO_2_ on root elongation in barley seedlings. However, this did not occur in seedlings treated with *n*CeO_2_ at the highest concentration, in which the root length was very similar to controls. In addition, in this case the literature reports contradictory evidence. López-Moreno et al. (2010a) reported that the root growth in maize and cucumber seedlings was significantly promoted by *n*CeO_2_ (up to 4000 mg l^-1^) whereas the same treatments resulted in a negative effect on root development in alfalfa and tomato. An inhibitory effect of *n*TiO_2_ on root elongation in cucumber was reported by Mushtaq (2011). A decrease in the number of secondary lateral roots in pea seedlings was verified by Fan et al. (2014), whereas Boonyanitipong et al. (2011) did not record any effect on root length in rice seedlings exposed to *n*TiO_2_. In our case, the different effect of the *n*CeO_2_ and *n*TiO_2_ on the root elongation is likely due to their different grade of agglomeration demonstrated by the z-average size and PDI values of *n*TiO_2_ that results significantly higher than *n*CeO_2_.

It might happen that the quantification analysis of trace metals in plant roots is disturbed by external contamination. In this case, the concentration of the element in the plant tissues could be significantly overestimated due to a fraction of metal, which is not taken up but simply adsorbed onto the external root surface. In our experiment, a concentration of Ce about 60 times greater than Ti, was found in barley root tissues. This substantial difference indicates that the procedures for preparation of the samples were conducted properly; otherwise, we would also have very high concentrations also for Ti.

Our results showed that the exposure of *H. vulgare* to *n*CeO_2,_ which are smaller and less aggregated than *n*TiO_2_, resulted in a greater total Ce concentration in roots compared to Ti. In can therefore be assumed that, for some still unknown reasons, the model of root uptake of the two elements could differ, depending in part on the intrinsic properties of solubility and agglomeration properties of *n*CeO_2_ and *n*TiO_2_. On the other hand, this is in agreement with the findings by Zhang et al. (2011), who verified that cucumber roots absorbed higher amounts of 7 nm *n*CeO_2_ than 25 nm ones. On the other, some studies pointed out the possibility of interactions between the root metabolism and MeNPs. Lin and Xing (2007) demonstrated that root exudates such as proteins, phenolic acids, and aminoacids have a role in the adsorption of ZnO NPs to the root surface of perennial rye-grass. More recently, Schwabe et al. (2015) observed that root uptake of dissolved Ce^(III)^ was promoted by the dissolution of *n*CeO_2_ at the medium-root interface in hydroponically growth sunflower and maize. A further confirmation about the role of root exudates on the adsorption of MeNPs was provided by Lv et al. (2015) and Ma et al. (2015), respectively, for *n*CeO_2_ and *n*ZnO, respectively. However, Lv et al. (2015) reported that a possible access of *n*ZnO to the root tissues could be through the root apex or the meristematic zone to the lateral root system where the Casparian strip is not yet developed.

Root-to-shoot translocation of *n*CeO_2_ has been previously described in soybean (Priester et al., 2012), tomato (Wang et al., 2012), cucumber (Zhao et al., 2013), and cotton (Van Nhan et al., 2015) after treatments with *n*CeO_2_ suspensions. Different observations have been made on *n*CeO_2_ root-to-shoot translocation in graminaceous crops. Schwabe et al. (2013) reported that wheat does not translocate *n*CeO_2_ into the aerial tissues, whereas Rico et al. (2013, 2015a) reported the translocation of *n*CeO_2_ from roots to rice grains and maize kernels, respectively. According to Rico et al. (2015b), we report evidence of Ce translocation from roots to the aerial part of barley. As regards Ti uptake and translocation, fewer data are available in the literature compared. However, our data are consistent with the findings reported by Song et al. (2013b) on tomato seedlings exposed to Ti at concentrations ranging from 50 to 5000 mg l^-1^.

Finally, we reported that root length in barley seedlings treated with 500 mg *n*CeO_2_ l^-1^ was significantly shorter than controls. This apparent dose-effect was not confirmed at higher *n*CeO_2_ concentrations, since the root length was similar to that of controls. Similar evidence was reported by López-Moreno et al. (2010a). According to Nascarella and Calabrese (2012) and Bell et al. (2014), such unexpected results might be interpreted as a hormetic effect of *n*CeO_2_ on root elongation in barley seedlings.

### Plant Stress Induced by Nanoparticle Treatments

Within the plants, NPs may interact with the host cells, causing different effects, ranging from cell death (if the host is sensitive) to not relevant cell modifications (in the case of host tolerance), depending on their type, shape, and concentration (Rico et al., 2011; Gardea-Torresdey et al., 2014). The microscopic observations on barley seedlings indicated that both *n*CeO_2_ and *n*TiO*_2_*, at the used concentrations used, were able to enter the root tissues, being detected in the parenchymal cells and xylem vessels. Even though we did not observe Ce and Ti crystalline aggregates in the shoots, ICP analyses suggested a root-to-shoot mobilization of Ce and Ti ions. At histological level the accumulation of such elements induced limited injuries. On the contrary, important differences in the effects of treatments were obtained at nuclear level, where only the *n*CeO_2_ treatments induced visible modifications in the chromatin aggregation in the nuclei of root and shoot parenchymal cells.

Condensed chromatin and fragmented nuclei are described as part of the programmed cell death (PCD), occurring in response to different environmental stimuli and stresses, induced by pathogens (Lam et al., 2001) and by diverse abiotic factors (White, 1996; Kratsch and Wise, 2000) including the exposition to nanomaterials (Shen et al., 2010). PCD plays an important role in mediating plant adaptation to the environment. In cells that undergo programmed death, chromatin condenses into masses with sharp margins, and DNA is hydrolyzed into a series of fragments (Gladish et al., 2006). Dynamic compaction of chromatin is an important step in the DNA-damage response, because it activates DNA-damage-repair signaling (Burgess et al., 2014) in response to injuries.

The hypothesis of Ce-induced DNA damage in treated seedlings finds further support in the results obtained with the RAPD test. RAPD can potentially detect a broad range of DNA damage and mutations, so it is suitable for studying MeNPs genotoxicity (Atienzar and Jha, 2006). The RAPD modified patterns at high concentrations of *n*CeO_2_ (1000–2000 mg l^-1^) indicated a genotoxic effect, which could directly influence the cell cycle. This is further confirmed by the reduced mitotic index recorded in the samples treated with *n*CeO_2_ 2000 mg l^-1^, which clearly demonstrated the negative effect of high *n*CeO_2_ concentrations on the cell cycle. Our results are in agreement with López-Moreno et al. (2010b), who demonstrated *n*CeO_2_ genotoxicity on soybean plants subjected to treatments similar to those reported in our work.

It is still far too early to conclude if the observed effects were direct or indirect consequences of the treatments, since *n*CeO_2_ were not found in the nucleus. As it is known that increasing oxidative stress leads to DNA damage, the higher presence of ROS in treated samples could cause modification in RAPD patterns. However, as our analysis on ROS indicated a peak at 500 mg *n*CeO_2_ l^-1^, it can be rationalized that lower concentrations triggered an initial oxidative signal, while only higher *n*CeO_2_ doses were able to induce damage at nuclear level. The oxidative stress peak at 500 mg l^-1^ dose and could be rationalized by the well-known SOD mimetic activity attributable to *n*CeO_2_, which could cause a dismutation of superoxide anions into H_2_O_2_. Since a similar pattern is also found for ATP measured in *n*CeO_2_ treated tissues, it is suggested that the oxidative burst induced by the more effective dose of *n*CeO_2_ could be associated to a stimulation of cellular respiration and a consequent increase in ATP production. This could be due to a defense response signal or an increased requirement for energy (Vranová et al., 2002).

On the contrary, the *n*TiO_2_ treatments did not influence either the mitotic index or RAPD pattern. This is in contrast to Moreno-Olivas et al. (2014) who observed *n*TiO_2_-induced genotoxicity in hydroponically cultivated zucchini. As the size of *n*TiO_2_ they reported is comparable to that used in our work, the different results obtained can be explained by (i) the different cultivation systems (Petri dishes vs. full nutrient solution in hydroponics) and (ii) the *n*TiO_2_ concentration used by Moreno-Olivas et al. (2014; 10-fold smaller). The latter potentially prevents the formation of big NP agglomerates, making them more bioavailable.

## Conclusion

Although investigations into the effects of NPs in plants continue to increase, there are still many unresolved issues and challenges, in particular at the biota-nanomaterial interface (Nowack et al., 2015). In this multidisciplinary work, we studied the phytotoxic and genotoxic impact of *n*CeO_2_ and *n*TiO_2_ cerium and titanium oxide NP suspensions on the early growth of barley. Seed germination was not affected by the *n*CeO_2_ and *n*TiO_2_ suspensions, indicating that *n*CeO_2_ and *n*TiO_2_ are not allowed to enter the seed coatings. However, we verified that the concentration of Ce and Ti in the seedling fractions, as well as the root-to-shoot translocation, were dose-dependent. Then, we found signals of genotoxicity (RAPD banding patterns and mitotic index) and phytotoxicity in root cells (oxidative stress and chromatin modifications) resulting in a shortage of root elongation.

The different magnitude of bioaccumulation of Ce and Ti suggests a different uptake mechanism, likely due to the different behavior of *n*CeO_2_ and *n*TiO_2_. Recent studies have shown that plant toxic effects of nanomaterials are not merely due to the particle size and concentration of a suspension. Phytotoxicity of metal oxide NPs is related both to the direct adsorption of particles onto the root structures and to the aptitude of the metal ion to dissolve, possibly mediated by binding molecules produced by plants in the medium-root interface.

Our study had not the objective to investigate the details of the mechanisms by which the NPs entering within the roots. However, we verified the presence of both *n*CeO_2_ and *n*TiO_2_ into the root cells where an increase in oxidative stress occurred. More research needs to be conducted to verify whether germination can be affected by smaller *n*CeO_2_ and *n*TiO_2_. In addition, we need to understand if modification of the physical–chemical properties of NPs at the root interface can foster the plant uptake of Ce and Ti forms.

## Author Contributions

AM conducted the experiments. AF and AB provided the biochemical parameters. FP performed out the ICP and RAPD analysis. RM made TEM observation and observed MeNPs distribution *in planta*. CG and MS carried out TEM–EDAX observations. MV contributed to the mitotic index. LM designed, coordinated the study, performed statistical analysis, and prepared the figures. All authors were involved in manuscript writing. All authors contributed to the revision of the manuscript.

## Conflict of Interest Statement

The authors declare that the research was conducted in the absence of any commercial or financial relationships that could be construed as a potential conflict of interest.
